# Extremely halophilic archaeal communities are resilient to short‐term entombment in halite

**DOI:** 10.1111/1462-2920.14913

**Published:** 2020-01-14

**Authors:** Tom J. C. Huby, Dave R. Clark, Boyd A. McKew, Terry J. McGenity

**Affiliations:** ^1^ School of Life Sciences University of Essex Colchester Essex UK

## Abstract

Some haloarchaea avoid the harsh conditions present in evaporating brines by entombment in brine inclusions within forming halite crystals, where a subset of haloarchaea survives over geological time. However, shifts in the community structure of halite‐entombed archaeal communities remain poorly understood. Therefore, we analysed archaeal communities from *in situ* hypersaline brines collected from Trapani saltern (Sicily) and their successional changes in brines versus laboratory‐grown halite over 21 weeks, using high‐throughput sequencing. Haloarchaea were dominant, comprising >95% of the archaeal community. Unexpectedly, the OTU richness of the communities after 21 weeks was indistinguishable from the parent brine and overall archaeal abundance in halite showed no clear temporal trends. Furthermore, the duration of entombment was less important than the parent brine from which the halite derived in determining the community composition and relative abundances of most genera in halite‐entombed communities. These results show that halite‐entombed archaeal communities are resilient to entombment durations of up to 21 weeks, and that entombment in halite may be an effective survival strategy for near complete communities of haloarchaea. Additionally, the dominance of ‘halite specialists’ observed in ancient halite must occur over periods of years, rather than months, hinting at long‐term successional dynamics in this environment.

## Introduction

Hypersaline ecosystems are diverse and globally distributed. Surface environments with >20% salinity and subjected to periodic halite precipitation are usually dominated by haloarchaea, which is the informal name for Archaea in the class *Halobacteria* (McGenity and Oren, 2012). Haloarchaea have a competitive advantage over many other halophiles at such high salinities, mainly because they adopt the ‘salt‐in strategy’, with KCl as the primary osmolyte, as opposed to the more energetically costly biosynthesis of organic osmolytes (Oren, 2011). However, they have numerous other adaptations that allow them to grow in the presence of multiple other stressors such as high temperatures, UV radiation and evaporation (Jones and Baxter, 2017). Coastal solar salterns have commonly been used to investigate successional community changes as salinity increases (Benlloch *et al*., 2002), as well as the environmental and biogeographic factors influencing microbial communities in hypersaline brines (Fernández *et al*., 2014) and halite crystals (Clark *et al*., 2017).

Halite precipitates over a range of salinities, and at the terminal stages, the remaining bittern brine becomes enriched in ions such as magnesium, the chaotropic nature of which leads to denaturation of biological macromolecules, thus restricting microbial activity (Javor, 1984; Hallsworth *et al*., 2007). In order to escape such chaotropic conditions or complete desiccation, haloarchaea (and other extreme halophiles) become entombed within brine inclusions of halite (Norton and Grant, 1988). When the halite dissolves, for instance after rainfall, the haloarchaea are released back into the brine whereupon they can resume normal growth. For example, experimental pure‐culture studies showed that all of the 14 haloarchaeal strains entombed in halite for 6 months were able to grow (Norton and Grant, 1988; Gramain *et al*., 2011). Brine inclusions within halite crystals can be highly abundant, up to 10^10^ cm^−3^ (Roedder, 1984). Within these brine inclusions, microorganisms may meet their metabolic needs by recycling organic molecules from necromass (McGenity *et al*., 2000; Thomas *et al*., 2019) or compounds leaked from other microbial cells, such as glycerol (Schubert *et al*., 2009; Oren, 2017). Interactions between entombed microbes are evidenced by the improved recovery of the haloarchaeon *Haloquadratum walsbyi* when co‐entombed with the Bacteroidetes species *Salinibacter ruber* compared with when entombed alone (Gramain *et al*., 2011). Additionally, some haloarchaea minimize their cell size to reduce metabolic requirements (Fendrihan *et al*., 2006; Winters *et al*., 2015; Jaakkola *et al*., 2016). Survival within halite crystals may enable some haloarchaea to survive over geological time (McGenity *et al*., 2000; Fendrihan *et al*., 2006; Lowenstein *et al*., 2011; Jaakkola *et al*., 2016), potentially making halophilic Archaea the oldest living organisms on Earth as well as leading candidates for astrobiological studies (Fendrihan *et al*., 2009). Notably, many studies of entombed archaeal communities from ancient halite show a reduction in community diversity compared to contemporary brine communities, suggesting that not all haloarchaea survive well in halite (Gramain *et al*., 2011). Furthermore, studies of marine‐derived halite crystals that are a few years old show that dominant members of brine communities are present in reduced abundance, or undetectable, in halite communities, whereas others that are scarce in brines are overrepresented in halite (Henriet *et al*., 2014; Clark *et al*., 2017). *Halobacterium* and *Halolamina* are examples of genera with species that are overrepresented in both culture‐dependent and culture‐independent, studies of commercial sea salt (Henriet *et al*., 2014; Koh *et al*., 2015; Clark *et al*., 2017; Gibtan *et al*., 2017) and ancient halite (McGenity *et al*., 2000; Mormile *et al*., 2003; Gramain *et al*., 2011; Jaakkola *et al*., 2016).

**Fig 1:**
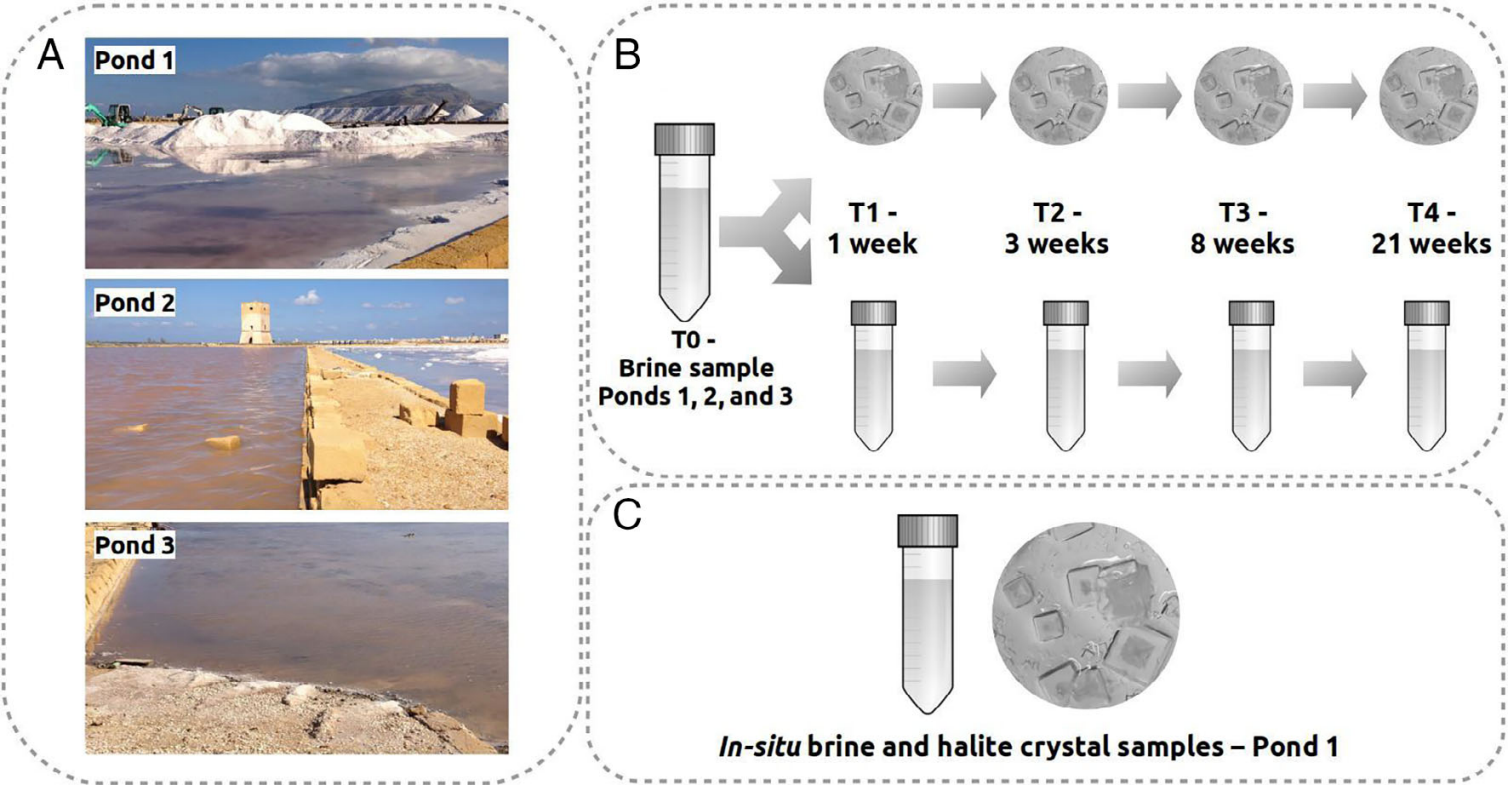
A. Views of the three sampled ponds at Trapani saltern, Sicily. B. Schematic view of the laboratory‐based succession experiment in which brine samples from each of the ponds were allowed to form halite crystals that were then sampled at 1, 3, 8 and 21 weeks. Comparisons of archaeal communities were made through time, and between halite and brine samples of the same age. C. For the additional analysis of *in situ* archaeal communities, brine and halite samples were collected from pond 1 only.

In order to address the gap in our understanding of the succession of natural communities within halite over several weeks after entombment, we used brine samples from three solar saltern crystallizer ponds in Trapani (Sicily, Fig. 1; Fig. S1), allowed halite to form in the laboratory and analysed archaeal communities over 21 weeks in halite crystals and the corresponding brines. Additionally, we compared the *in situ* brine and halite communities from one of the ponds. We hypothesized that, over the 21‐week experiment: (i) the abundance of Archaea in halite would decrease through time, and in comparison to brine controls, (ii) halite communities would become less diverse and more compositionally distinct through time for all three brines as the community is reduced to specialist ‘halite survivors’ and (iii) archaeal taxa would show differential changes in abundance, with halite specialists increasing in abundance through time.

## Results

### Ionic and microbial composition of in situ and parent brines

Figure 1 shows the overall study design, and Table 1 shows the water activity (*a*_w_), refractive index and ionic composition of the brines from the three different evaporation ponds. Brine 1 was from a crystallizer pond where the salt had been harvested immediately before sampling and had the lowest water activity (0.716) of the three ponds. This was followed by brine 3 (0.734), which was also a recently harvested crystallizer pond. Brine 2 had the highest water activity of the three samples at 0.755 and was derived from an evaporation pond approaching saturation. The brine of pond 1 had the highest Mg^2+^ concentrations, followed by brine 3, and brine 2, while the Na^+^ concentration showed the opposite trend.

**Table 1:** Major ion concentrations (g l^−1^) and water activity (*a*_w_) of the brines derived from three hypersaline saltern ponds, and used in the laboratory succession experiment.

Ion	Ponds from which the brines derived		
	1^a^	2	3
Na^+^	82.5	116.7	100.9
Mg^2+^	42.3	16.8	24.2
Ca^2+^	0.2	0.1	0.2
K^+^	11.8	5.9	6.8
Li^+^	0.002	0.001	0.002
Cl^−^	198.9	200.4	193.5
SO_4_^2−^	59.1	19.8	32.4
Refractive index	1.388	1.379	1.383
Water activity (*a*_w_)	0.716	0.755	0.734

a Pond 1 was also sampled for the *in situ* comparison of brine and halite.

Out of 87 samples (*in situ* and laboratory experiment), seven did not yield archaeal amplicons. Sequences from successfully amplified samples were clustered into operational taxonomic units (OTU) at the 97% similarity level. OTUs were removed if they: identified as chimeras (*de novo* or reference‐based), were <200 bp, or were singletons. After the removal of OTUs and individual samples based on the above criteria a total of 1,715,018 archaeal sequences remained from 75 samples. These sequences represented a total of 277 OTUs prior to rarefaction, 77.6% of which were assigned to the class Halobacteria.

Taxonomic analysis of the Archaea from *in situ* brine and halite samples collected from pond 1 revealed that communities were dominated by haloarchaea as expected (Fig. S2). Archaea from the orders Haloferacales and Halobacteriales accounted for 96.6% of the *in situ* brine community and 98.3% of the *in situ* halite‐entombed communities, with the genus *Halorubrum* alone accounting for >75% of the total halite‐entombed community. Notably, *Halorubrum* was one of a few genera that were relatively more abundant in the *in situ* halite‐entombed community compared with the brine, whereas *Haloquadratum*, *Halorientalis* and *Halomicrobium* showed the reverse trend, being relatively more abundant in the brine. *In situ* brine communities were more OTU rich than the *in situ* halite communities with 60–64 OTUs per sample compared to 40–56 OTUs in the halite.

### Changes in the abundance of halite‐entombed communities through time

**Fig 2:**
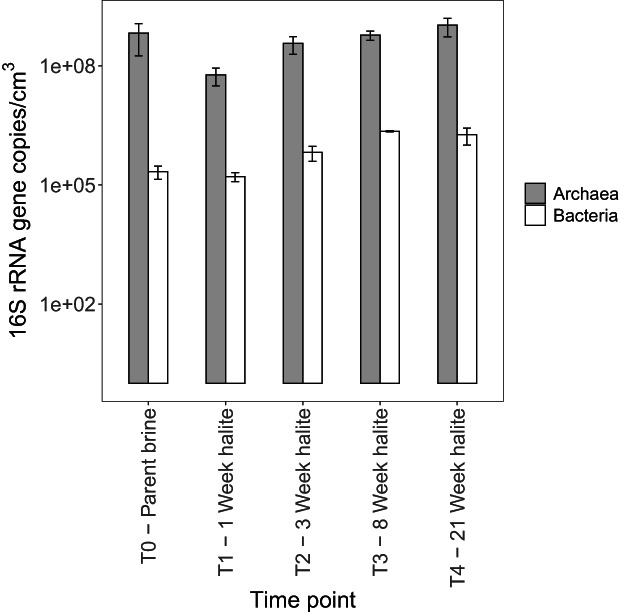
Quantitative PCR analysis of archaeal and bacterial 16S rRNA genes, showing that archaeal 16S rRNA genes were several orders of magnitude more abundant than those from Bacteria in the brines and halites analysed in this study. Furthermore, the total abundance of archaeal 16S rRNA genes did not show any clear temporal trend over 21 weeks. Abundances are averaged over samples derived from all three ponds. See Experimental Procedures for a description of how values were normalized to account for the different physical state of brine and halite.

Quantitative PCR analysis of archaeal and bacterial 16S rRNA genes in the halite samples compared with the parent brines revealed that across the duration of the succession experiment, archaeal gene copies were relatively more abundant, is approximately 530 times more abundant than those of Bacteria on average (coef = 534.96, *z* = 11.52, *p* < 0.001), and so we focussed on Archaea for further community composition analyses (Fig. 2). Throughout the course of the experiment, the total abundance of archaeal 16S rRNA gene copies was remarkably stable (Fig. 2). Aside from the 1‐week‐old halite (T1), variations in archaeal abundance from the parent brine were statistically unclear (T2; coef = 0.56, *z* = −0.95, *p* = 0.34, T3; coef = 0.90, *z* = −0.18, *p* = 0.86, T4; coef = 1.59, *z* = 0.75, *p* = 0.45), suggesting that halite‐entombed Archaea were resilient over the short term. In contrast to other time points, there was a small but clear reduction in archaeal 16S rRNA gene abundance in the 1‐week‐old halite compared to the parent brine (coef = 0.09, *z* = −3.93, *p* < 0.001).

### Compositional dynamics of Archaeal halite‐entombed communities

**Fig 3:**
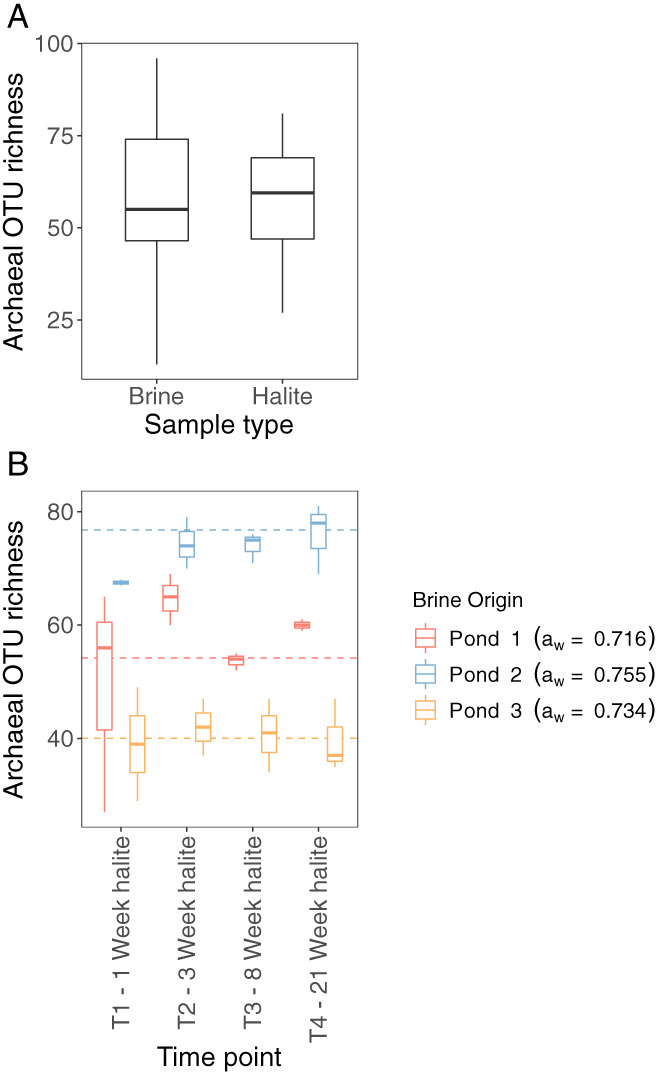
OTU richness, derived from 16S rRNA gene sequences, of (A) halite‐entombed archaeal communities compared to brine controls from the succession experiment, and (B) of halite‐entombed archaeal communities derived from each of the three ponds sampled over the 21 week duration of the succession experiment. Dashed lines indicate the mean OTU richness across all time points for halite‐entombed communities from each of the three parent brines.

Amplicon sequencing of archaeal 16S rRNA genes revealed that throughout the course of the experiment, there was no clear difference in OTU richness between halite‐entombed communities and brine controls (Fig. 3A, coef = 0.95, *z* = −0.61, *p* = 0.55). Furthermore, in contrast to hypothesis 2, there was no clear decline in archaeal OTU richness in halite and brine communities through time (coef = 1.01, *z* = 1.32, *p* = 0.19), nor was there any evidence of interaction between community type (brine versus halite) and time (coef = −0.01, *z* = −0.83, *p* = 0.41). By focussing on the halite communities, we observed clear differences in OTU richness between communities originating from different brines (Fig. 3B), with communities originating from pond 2 (with the highest *a*_w_) being more OTU rich than those in pond 1, which has the lowest *a*_w_ (coef = 1.41, *z* = 4.43, *p* < 0.001), and communities derived from pond 3 (with intermediate *a*_w_) being less OTU rich than those of pond 1 (coef = 0.79, *z* = −2.67, *p* < 0.01). Again, there was no clear evidence of temporal changes in richness across all brines (Fig. 3B, coef = 1.00, *z* = 0.81, *p* = 0.42), or interactions between brine origin and time (brine 2 × time; coef = 1.00, *z* = −0.72, *p* = 0.47, brine 3 × time; coef = 0.99, *z* = −0.76, *p* = 0.45). In fact, OTU richness in 21‐week‐old halite crystals was almost identical to the richness observed in 1‐week‐old halite regardless of brine origin, from which it can be inferred that archaeal diversity remains stable in halite‐entombed communities over short time scales (<5 months) and that this pattern holds across brines of different ionic composition.

**Fig 4:**
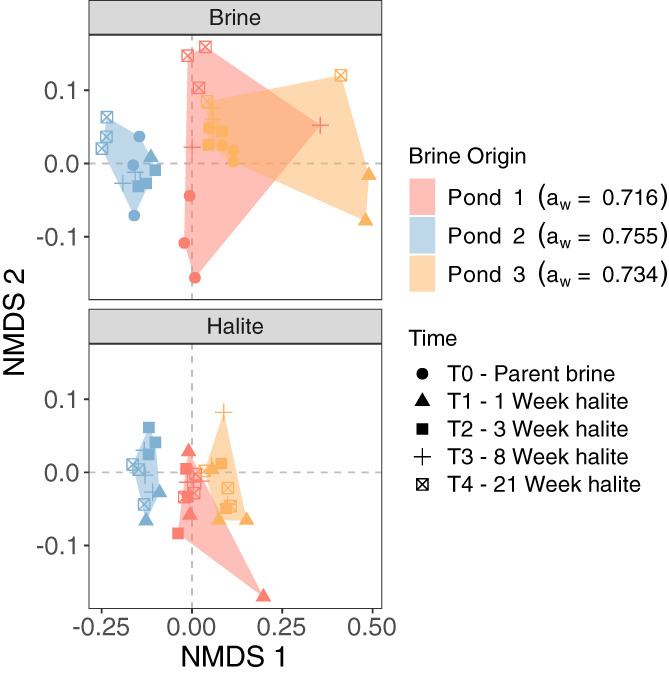
Non‐metric multidimensional scaling (NMDS) analysis of archaeal communities, based on Sørensen dissimilarity (using OTUs derived from 16S rRNA gene sequences). Each point represents a single community, with points closer together indicating compositionally similar communities. For ease of visualization, halite and brine control communities from a single NMDS analysis have been split into separate panels. Convex hulls highlight the spread of communities originating from the same parent brine.

As with OTU richness and population size, changes in archaeal community composition over 21 weeks of entombment in halite were more subtle than expected. PERMANOVA analysis revealed clear, but very small, compositional differences between halite‐entombed and brine‐control communities (pseudo‐*F*_1, 61_ = 2.40, *R* ^2^ = 0.03, *p* < 0.05) and through time (pseudo‐*F*_4, 59_ = 1.94, *R* ^2^ = 0.11, *p* < 0.01). When analysing only halite communities, the influence of time on archaeal community composition was unclear (pseudo‐*F*_3, 23_ = 1.04, *R* ^2^ = 0.06, *p* = 0.43), whereas brine origin had a much more pronounced effect (Fig. 4, pseudo‐*F*_2, 23_ = 8.13, *R* ^2^ = 0.34, *p* < 0.001). Together, these results suggest that the composition of halite‐entombed archaeal communities is much more strongly related to the brine from which the halite was derived than to the duration of entombment, at least over the timescale measured here.

### Taxonomic composition of halite‐entombed communities

**Fig 5:**
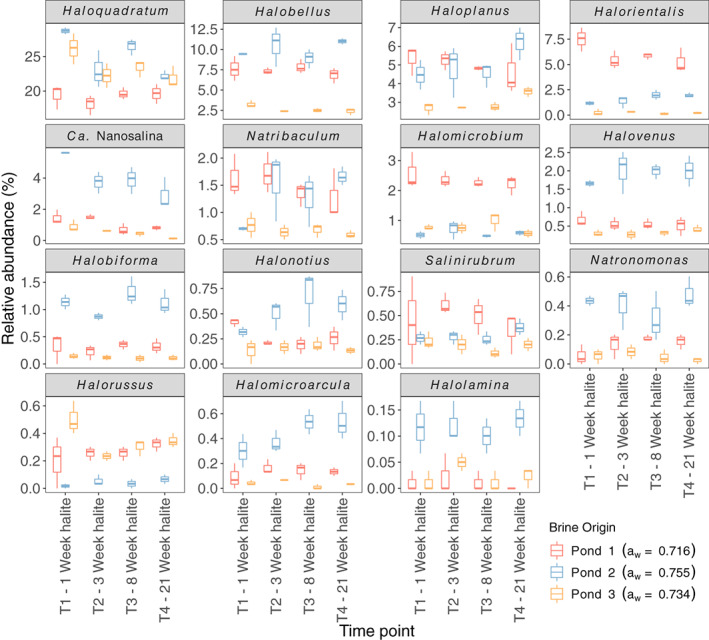
The relative abundances of archaeal genera in halite, for which parent brine was a statistically important predictor. Only genera whose average relative abundance, based on 16S rRNA gene analysis, was >0.1% are shown, and genera are arranged in order of decreasing median relative abundance across the succession experiment.

After aggregating OTUs within each identified genus, multivariate‐GLMs (MV‐GLMs) revealed that time alone had a statistically clear effect on eight genera across sample types and parent brines (Fig. S3 and Table S1). Of these genera, few displayed any clear temporal changes in relative abundance within the halite, but instead, the detected temporal effects were driven by changes in abundance in brine controls. For example, both *Halobacterium* and *Halolamina*, showed a statistically clear increase in abundance in brines. *Halobacterium* increased from 0.004% at T0 to 0.57% at T4 (coef = 1.21, *deviance* = 11.60, *p* < 0.01), while *Halolamina* increased in abundance in the brine from 0.06% at T0 to 1.01% at T4 (coef = 1.08, *deviance* = 14.80, *p* < 0.01). The exception to this was *Candidatus* Nanosalina, which decreased in relative abundance through time in both brine controls and halite samples (coef = 0.99, *deviance =* 6.13, *p* < 0.05). In contrast, brine origin was a statistically important predictor of relative abundance for 22 of the 30 archaeal genera analysed. Notably, many of these genera were most abundant in halite crystals from pond 1 (lowest *a*_w_) or pond 2 (highest *a*_w_), with relatively few genera being most abundant in halite derived from pond 3, the parent brine of intermediate *a*_w_ (Fig. 5). *Halorubrum*, the most abundant genus overall, with a mean relative abundance of 55.2%, showed no clear differences in abundance between the three brines.

## Discussion

We characterized the structure of archaeal communities entombed in laboratory‐grown halite crystals over 21 weeks. Our experiment revealed that archaeal communities are surprisingly resilient to entombment over this time scale. Against expectations, archaeal population sizes (based on qPCR) did not decrease over time, and were not clearly different after 21 weeks of entombment from the start. Furthermore, archaeal diversity remained stable, suggesting that a large proportion of the brine community are able to become entombed and were still detectable after 21 weeks. Finally, we found that the period of entombment did not appear to be an important determinant of the abundance of specific haloarchaeal genera within the halite communities. In contrast, brine origin played a major role in determining the abundance of specific genera, further suggesting that the parent brine is the major driver of archaeal community structure in these halite crystal environments, at least over short (<5 months) timescales.

### Archaeal communities are resilient to short‐term halite entombment

Despite the poly‐extreme conditions present in many hypersaline environments, halophilic archaeal communities are usually highly diverse. However, analyses of ancient halite communities from a variety of geological horizons have revealed limited diversity, often with only a few dominant genera present (McGenity *et al*., 2000; Mormile *et al*., 2003; Schubert *et al*., 2009; Gramain *et al*., 2011; Jaakkola *et al*., 2016). The difference in diversity between modern brine and ancient halite communities implies that most haloarchaea are not capable of long‐term survival in halite. However, the timescale over which this decline in diversity and population size occurs is not well understood. Our results show that, over a period of 21 weeks, entombed haloarchaeal communities are resilient, with few clear temporal trends in either diversity or abundance. In this respect, our results agree with those of Clark and colleagues (2017), who showed that sample age did not correlate with archaeal OTU richness in a series of contemporary halite samples representing ages from approximately 4–10 years. However, the halite samples analysed by Clark and colleagues (2017) also derive from a variety of different brines. Here, we show that instead of time, parent brine composition is a major determinant of archaeal diversity, thus the results are not directly comparable. Our results therefore suggest that regardless of parent brine, the decline in diversity and population size associated with long‐term entombment in halite is likely to happen over a timescale of several years rather than months. This observation is consistent with the known slow growth of haloarchaea in crystallizer ponds, with doubling times of between 2 and 50 days (Pedrós‐Alió *et al*., 2000). Thus, any increase in the relative abundance of haloarchaeal taxa that are better adapted than others to live inside halite (see below) may require a considerable period of time to manifest itself.

### Long‐term‐survival specialist genera, *Halobacterium* and *Halolamina* were enriched over time, but only in the brines

It is notable that two of the haloarchaeal genera that increased in relative abundance in the brine samples over time were *Halobacterium* (by ~two orders of magnitude) and *Halolamina* (by ~one order of magnitude), both of which are typically enriched in halite crystals, especially those that are several years old (Henriet *et al*., 2014; Koh *et al*., 2015; Clark *et al*., 2017; Gibtan *et al*., 2017), through to geological samples of tens of thousands to millions of years old (McGenity *et al*., 2000; Mormile *et al*., 2003; Gramain *et al*., 2011; Jaakkola *et al*., 2016). We can speculate that this enrichment of *Halobacterium* and *Halolamina* in the brines from a low baseline abundance is a reflection of their capacity to preferentially survive in systems that are not replenished with nutrients. The higher level of aeration in the brines compared to halite crystals, and thus more rapid multiplication, may be a factor that led to the observed change in these genera in the brines but not in the halite. It will be intriguing to extend the succession experiment to see whether this trend continues in the brines, and whether these genera start to increase in relative abundance in the halite samples.

### The DPANN halophile Candidatus Nanosalina decreased in abundance in both halite and brines

A non‐haloarchaeal taxon became less abundant in halite (as well as brine) over time, namely, *Ca*. Nanosalina, which belongs to the Nanohaloarchaeota, part of the DPANN supergroup of Archaea (Rinke *et al*., 2013). This taxon has previously been recorded in halite crystals of between 4 and 10 years old (Clark *et al*., 2017) at relatively low abundance (0%–8% of the archaeal community) and many other hypersaline environments, and its physiology has been inferred using genomes assembled from metagenomes (Ghai *et al*., 2011; Narasingarao *et al*., 2012; Podell *et al*., 2014; Di Meglio *et al*., 2016). While it was suggested by Narasingarao and colleagues (2012) that their small physical size may enable them to remain suspended in oxygenated surface waters and thus cater for aerobic metabolism, Andrade and colleagues (2015) suggest that Nanohaloarchaeota do not have genes encoding cytochrome C oxidase, and are therefore unable to use oxygen. The proposal that Nanohaloarchaeota have heterotrophic fermentation‐based metabolism and are dependent on metabolites from other organisms (Andrade *et al*., 2015) was recently supported by co‐culture of *Ca*. Naohaloarchaeum and the haloarchael species *Halorubrum lacusprofundi* (Hamm *et al*., 2019). However, the Nanohaloarchaeota are phylogenetically broad, and collectively are likely to demonstrate a wide variety of interactions and physiological activities. It is therefore too early to speculate on the reasons for their relative decline over time. However, it may be that they and/or their hosts have a competitive advantage in a more natural fluctuating environment (Andrade *et al*., 2015).

### The parent brine was the main factor in determining the relative abundance of most genera in halite‐entombed communities

The observed dominance of haloarchaea and the types and abundance of the genera found is typical of saltern crystallizer ponds (Ventosa *et al*., 2015). The small but clear differences in archaeal community composition between the three brines likely reflects differences in the brines’ ionic composition and water activity, rather than, for instance, dispersal limitation, given the short distance and connectivity of the ponds. Such physicochemical parameters influence microbial (including haloarchaeal) growth (Hallsworth *et al*., 2007; Stevenson *et al*., 2015; Fox‐Powell *et al*., 2016), and are major drivers for the dominance of genera such *Haloquadratum* and *Halorubrum* in crystallizer ponds (Podell *et al*., 2014), several representatives of which have been shown to grow at very low water activity in the presence of relatively high concentrations of magnesium ions (Oren, 1983; Bolhuis *et al*., 2004, 2006; Burns *et al*., 2007; Hallsworth *et al*., 2007). In this case, it is not known whether the ionic composition directly or indirectly influences the archaeal community. Indirect effects may include salinity‐induced differences in microbial populations not investigated here that could affect the archaeal community, such as benthic microbes that may supply organic matter. We can rule out the effect of the planktonic glycerol‐producing chlorophyte, *Dunaliella salina*, which is often the dominant primary producer in salterns and occasionally in crystallizer ponds (Oren, 2005), as no *Dunaliella* chloroplast 16S rRNA sequences were detected in our study (bacterial 16S rRNA amplicon sequence data not shown), consistent with studies from some other crystallizers (Pedrós‐Alió *et al*., 2000).

Overall the minimal temporal change in halite‐entombed archaeal communities irrespective of the brine from which they were derived, suggests that the ionic composition of the parent brine has no effect on the temporal dynamics of entombed communities, at least over the relatively short time frame of our experiment.

### In contrast to the laboratory succession experiment, there were some notable differences in situ between the halite entombed and brine communities

The *in situ* community analysis of pond 1 demonstrates a higher relative abundance of *Halorubrum* and lower abundance of *Haloquadratum* in halite compared with the brine. This observation is consistent with that of Baati and colleagues (2008, 2010), and initially seems inconsistent with findings from our succession experiment, in which there was little change in archaeal community composition from parent brine to 21‐week‐old halite. There are two possible explanations for this observation that are not mutually exclusive: (i) conditions in the field, notably natural Mediterranean light and wind‐driven aeration, compared with the laboratory, may have led to preferential exclusion or early loss of *Haloquadratum* spp. from halite *in situ*; and (ii) although *in situ* halite and brine samples were obtained at the same time, the halite crystals were formed from an earlier brine, which *inter alia* would have been less saline and thus may have had a different community. Variation in saltern community composition on this scale has been observed at intervals of months (Gomariz *et al*., 2015), but not, to the best of our knowledge, investigated over a few days, which would reflect the temporal difference in our study.

### How representative are 16S rRNA gene sequences of a viable archaeal community

It is pertinent to consider an alternative explanation for the lack of temporal dynamics observed here, especially given that DNA can stay intact in the environment, and particularly in hypersaline, anoxic environments (Borin *et al*., 2008). First, there was a decrease in relative abundance of *Candidatus* Nanosalina 16S rRNA genes, suggesting that death, cell lysis and degradation of DNA in the halite would probably have been observed in haloarchaeal taxa too, if it were occurring. Moreover, salterns are considered to be thermodynamically moderate environments, being well within the water‐activity limit of life and maintaining relatively high levels of activity (Stevenson *et al*., 2015; Lee *et al*., 2018), including DNA degradation, which is a widespread trait in haloarchaea (Oren, 2014a, b), as DNA can be used as a source of carbon, nitrogen and especially phosphorous (Chimileski *et al*., 2014; Zerulla *et al*., 2014). Moreover, our methodology involved collecting cells by centrifugation, and so any free DNA would have been lost at that stage.

Many haloarchaea are known to be polyploid, which can contribute to their capacity for long‐term survival, with the extra chromosomes providing a store of phosphorous (Chimileski *et al*., 2014; Zerulla *et al*., 2014) and serving as a template to allow faithful recombination after DNA damage (Soppa, 2013; Ludt and Soppa, 2019). However, polyploidy, along with gene copy number variation, can compromise absolute quantification of taxa when using genetic markers (Soppa, 2017), hence our focus on relative change, especially in halite over time, in 16S rRNA gene abundance. A change in ploidy in response to phosphorous limitation has been demonstrated in haloarchaea such as *Haloferax volcanii* (Zerulla *et al*., 2014). However, it is highly unlikely that the absence of change in haloarchaeal 16S rRNA gene abundance over time in halite was due to decreased ploidy from some taxa balanced by cell growth or increased ploidy in others (or *vice versa*). If this were the case, a concomitant change in community composition would have been observed.

It could be argued that the DNA came from intact but dead cells. However, representative species of many of the genera observed in our study have also been cultured from natural marine halite, e.g. *Haloarchaeum*, *Haloarcula*, *Halobaculum*, *Halobacterium*, *Halobellus*, *Haloferax*, *Halogranum*, *Halogeometricum*, *Halolamina*, *Haloplanus*, *Halorubrum*, *Halosimplex*, *Natronoarchaeum*, *Natronomonas*, *Salarchaeum*, *Salinarchaeum* (see summaries by Lee (2013), Henriet and colleagues (2014) and Bernard (2018)), and/or cultivated after laboratory entombment in halite, e.g. *Halorubrum*, *Halobacterium*, *Haloquadratum* (Norton and Grant, 1988; Gramain *et al*., 2011), thus demonstrating their potential to remain viable inside halite. Also, we obtained growth in Payne's liquid medium (Norton and Grant, 1988) from the halite used in this study, 1‐year after entombment, indicating that at least a subset of the entombed community was viable for a period beyond the duration of our 21‐week experiment.

### Implications of understanding halite‐entombed archaeal dynamics and concluding remarks

Understanding the dynamics of microbial communities in crystallizer ponds and halite crystals is important, as the carotenoid‐rich microbial community positively influences salt production and yield by enhancing light absorption and thus increasing the local temperature (Javor, 2002), and can even influence the form of halite crystals (Norton and Grant, 1988; Lopez‐Cortes *et al*., 1994). The fact that marine‐derived halite is laden with haloarchaea has important implications for sea‐salt applications. For example, haloarchaea derived from sea salt can contribute positively to diverse fermentation processes, such as fish‐sauce production (Thongthai *et al*., 1992; Lee, 2013) or lead to the spoilage of salted fish and hides (Lochhead, 1934). Thus, the results from this study combined with those of Clark and colleagues (2017), which collectively indicate that major haloarchaeal community changes in halite crystals occur over years rather than weeks, may guide how salt is stored and when it is applied in these commercial applications. Entombed microbes also alter the profile of volatile organic compounds (largely derived from carotenoids) in sea salt, which in turn will affect flavour as well as the capacity to identify the provenance of, for example, artisanal halite (Donadio *et al*., 2011). The potential influence of ingested haloarchaea (Oxley *et al*., 2010) on the health of humans and other animals remains to be discovered.

Halophilic microorganisms have become a key model in our quest to understand the potential for life on Mars due to their resistance to Martian‐like conditions such as high UV radiation and low water activity (Oren, 2014a, b). Mars harbours ancient deposits of various evaporitic minerals, potentially preserving extremophilic microorganisms from a time when the surface of Mars was more habitable (McLennan *et al*., 2005). Our results indicate that if this is the case, these communities are unlikely to represent the original source community, given the large time period that has passed. However, in areas of Mars where evaporite minerals may be periodically re‐dissolved, such as the possible sub‐glacial lakes recently identified (Orosei *et al*., 2018), evaporite‐entombed communities may persist that are more representative of the ion‐rich liquid water that once flowed over the Martian surface.

In conclusion, we reject our three hypotheses that, over 21 weeks: (i) the abundance of Archaea in halite would decrease through time, and in comparison to brine controls, (ii) halite communities would become less diverse and more compositionally distinct through time for all three brines and (iii) archaeal taxa would show differential changes in abundance in halite over time. The evolutionary advantage of halite entombment as a strategy to prevent desiccation or exposure to highly chaotropic brines is likely to be strong and consequently, is probably a universal feature of haloarchaea (but not of Nanohaloarchaeota). Moreover, the relatively slow growth of haloarchaea likely contributes to minimal detectable change in haloarchaeal community composition in halite over the 21‐week experiment. However, increased relative abundance of *Halolamina* and *Halobacterium* in the brines suggests that representatives of these two genera, which are often dominant in halite crystals >4 years old, become competitive in brines where nutrients are not replenished. Combining longer‐term investigations of natural communities with assays of the *in situ* activity and gene expression of model haloarchaea will yield further insight into the ecology and ecophysiology of haloarchaeal communities in halite and other evaporitic minerals.

## Experimental procedures

### Sampling

Samples were collected from Trapani Salterns, Sicily (37°58′49.9″N 12°29′42.0″E; Fig. S1), on 29 October 2016. For laboratory succession experiments, brines were collected in sterilized 1‐l Duran bottles from three saltern ponds (Figs. 1 and S1), and the salinity of each was determined using a hand‐held refractometer (BS Eclipse 45–41). For *in situ* community analysis, brine (10 ml) from three locations of pond 1 (a, b and c), approximately 5 m apart, was filtered through a 0.22 μm Sterivex filter (Millipore) using a 50‐ml syringe. Filters were stored in RNAlater (Qiagen). Halite crystals were collected in sterile Falcon tubes from the brine‐crystal interface at locations 1a, 1b and 1c. Filters and crystals were transported on dry ice and stored at −20°C upon return to the laboratory.

### Physical and chemical analysis of brines

The water activity of the three brines was measured at room temperature using an AW SPRINT 5000 (Novasina) as described in Hallsworth and colleagues (2007). The ionic composition of the three brines was analysed using a Dionex ICS‐3000 following the procedure described in Aslam and colleagues (2016). Prior to each run, brine samples were diluted 1000‐fold to a volume of 20 ml.

### Laboratory succession experiment

From each of the 1‐l brine samples from ponds 1, 2 and 3, 40 ml was transferred into 50 ml Falcon tubes in triplicate, and 20 ml aliquots of each sample were transferred to Petri dishes (10 cm diameter) in triplicate and left to evaporate in the ambient conditions of the laboratory. When halite crystals formed, they were transferred to sterile 50‐ml Falcon tubes using a spatula. Cells were collected from each brine before crystal formation (T0), and from brines and crystal samples after 1 week (T1), 3 weeks (T2), 8 weeks (T3) and 21 weeks (T4). This timescale is comparable to that which *in situ* archaeal communities might endure entombed in halite during a Mediterranean summer.

### DNA extraction

DNA was extracted from filtered *in situ* brine samples by ejecting RNAlater and aseptically removing the filter from the Sterivex casing. Each filter was cut into four pieces of equal size using a scalpel and placed into micro‐centrifuge tube containing 200 μl of autoclaved MilliQ water and vortexed briefly. The cell suspension/lysed cells (200 μl) and filter were transferred to bead‐beating tubes containing 0.5 g of 0.1‐mm diameter zirconia/silica beads (BioSpec) for DNA extraction. For the laboratory succession experiment, at each time point, cells from 3 ml of brines 1 and 3, and 4.5 ml of brine 2 were collected by centrifugation at 11 300*g* for 10 min. Cells from 5 g of halite crystals were collected by dissolving crystals in 20 ml of 10% NaCl, 1% MgSO_4_.7H_2_O. Tubes containing dissolved crystals were centrifuged at 10 000*g* for 40 min at 4°C. After centrifugation, the supernatant was removed, leaving approximately 5 ml. Cells were re‐suspended and transferred into 1.5 ml tubes for further centrifugation at 11 300*g* for 5 min prior to DNA extraction. MilliQ water (200 μl) was added to the cell pellet and transferred into 2‐ml bead‐beating tubes in preparation for DNA extraction. Cells from *in situ* halite crystals were collected in the same way as described above. DNA extraction was carried out according to the method in Griffiths and colleagues (2000).

### Quantitative PCR analysis

As a proxy for the total abundance of both Archaea and Bacteria, the abundance of archaeal and bacterial 16S rRNA gene copies in each sample was quantified by qPCR using the Archaea‐specific primers 344F and 915R (Stahl and Amann, 1991; Raskin *et al*., 1994) and Bacteria‐specific primers 341F and 534R (Muyzer *et al*., 1993). While qPCR analysis of 16S rRNA genes is prone to sources of bias due to polyploidy and multiple 16S rRNA gene copies per genome, here it provides a more robust alternative to estimating total microbial biomass than culture‐dependent methods such as counts of colony‐forming units, which are unreliable when many taxa are difficult to culture, or microscopy methods such as fluorescence *in situ* hybridization, which would be logistically challenging in halite‐entombed communities and difficult to scale up to large numbers of samples. All qPCRs were run against a standard curve, which was created from a purified PCR product from the same set of DNA extracts, quantified via a PicoGreen assay, and copy numbers calculated as described by McKew and Smith (2015). All qPCRs were performed in triplicate and consisted of 5 μl of SensiFAST™ SYBR® No‐ROX Mix (2X; Bioline), 0.2 μl of each primer (10 μM) and 1 μl of template DNA (total reaction volume of 10 μl). Thermocycling conditions consisted of an initial denaturation at 95°C for 3 min, followed by 39 cycles of 95°C for 10 s and 60°C for 30 s. Bacterial 16S rRNA genes were similarly amplified for 39 cycles, consisting of 95°C for 10 s and 60°C for 30 s, and included an initial denaturation step at 95°C for 3 min. A melt curve analysis was conducted at the end of the PCR to check the specificity of the qPCR reactions. Analysis of the standard curves from both qPCR assays showed high efficiency (efficiency >78.9%, *R* ^2^ *>* 0.99).

To account for differences between the physical states and densities of fluid brines and solid halite crystals, the 16S rRNA gene copy numbers derived from each sample were normalized to 1 cm^3^ of brine or halite. The density of saturated NaCl solution is 1.202 g/cm^3^ whereas the density of halite is 2.165 g cm^−3^. As 1 ml is equivalent to 1 g cm^−3^, the 16S rRNA gene copy number from 1 ml of saturated brine was divided by 1.202 g cm^−3^. Gene copy numbers from 1 g of halite were multiplied by 2.165 g cm^−3^.

### Sequencing library preparation

To characterize the archaeal communities of brine and halite samples, we sequenced the 16S rRNA gene using the Archaea‐specific primer pair 344F and 915R, where each primer was flanked by Illumina‐specific overhang sequences, as in Clark and colleagues (2017). Thermocycling was carried out as follows: 35 cycles (94°C for 15 s, 60°C for 15 s and 72°C for 15 s, with an initial denaturation step at 94°C for 3 min and a final elongation step of 72°C for 10 min). All initial PCRs were composed of 12.5 μl AppTaq Redmix (2X; Appleton Woods), 1 μl of each primer (10 μM), 1 μl of DNA extract and 9.5 μl of PCR water (25 μl total reaction volume). The presence and size of PCR products were checked by electrophoresis and ethidium‐bromide staining of 1% w/v agarose gels. PCR products were then purified, indexed and pooled as described previously (Clark *et al*., 2017). Briefly, PCR products were purified using AxyPrep™ Mag PCR Clean‐up beads (Appleton Woods Ltd.), using the Illumina protocol (https://support.illumina.com/downloads/16s_metagenomic_sequencing_library_preparation.html). Following purification, a short‐cycle, second‐stage PCR was carried out to barcode samples using Nextera XT indices (Illumina). Thermocycling used the following conditions: 95°C for 3 min, followed by 8 cycles of 95°C for 30 s, 55°C for 30 s and 72°C for 30 s, with a final elongation step at 72°C for 5 min. Indexed PCR products were again purified as described above, before being quantified using the Quant‐iT™ PicoGreen™ dsDNA Assay Kit (Invitrogen) according to the manufacturer's instructions on a FLUOstar Omega Microplate Reader (BMG Labtech). PCR products were then pooled in equimolar ratios, and the resulting pooled library was again quantified by the PicoGreen assay. The library was then sequenced on an Illumina MiSeq with V3 chemistry (2 × 300 bp paired‐end reads).

### Bioinformatics

Due to the length of the amplicon, pair‐end alignment of reads was not sufficiently reliable, and so the forward reads only were used in subsequent analyses, as in Clark and colleagues (2017). Bioinformatic analyses were then conducted, broadly following approaches described in Dumbrell and colleagues (2017). Sequences were first quality trimmed using Sickle (Joshi and Fass, 2011) with default settings, before error‐correction using the BayesHammer algorithm (Nikolenko *et al*., 2013) within SPADes (Nurk *et al*., 2013). Sequences were then de‐replicated and sorted by abundance with VSEARCH (Rognes *et al*., 2016) at a 97% similarity threshold. UCHIME, as implemented in VSEARCH, was used to identify both *de novo* and reference‐based (using the RDP database, release 11) chimera sequences, which were then removed from subsequent analyses (Edgar *et al*., 2011). Furthermore, any OTUs with centroid sequences <200 bp in length were discarded. Taxonomy assignment was performed with the RDP classifier (Wang *et al*., 2007). Raw sequence data have been submitted to the European Nucleotide Archive under the accession number PRJEB34255.

### Statistical analyses

Analyses were conducted on rarefied archaeal community data (1500 sequences per sample) using the vegan package (Oksanen *et al*., 2019) in R (R Core Team, 2019). To test whether archaeal population sizes decreased in halite through time (hypothesis 1), we tested for differences in archaeal 16S rRNA gene copy numbers across sampled time points using a negative‐binomial GLM (Venables and Ripley, 2002). For hypothesis 2, we tested for differences in OTU richness across sample types (halite versus brine) and parent brine (Ponds 1, 2, and 3), including an interaction with time point in both models, again using negative‐binomial GLMs. Community dissimilarity was quantified using the Sorensen index (Baselga and Orme, 2012). PERMANOVA analyses were used to test whether archaeal communities were more similar within time points, sample types or brine origins, than between them, using 1000 permutations (Anderson, 2001). Non‐metric multidimensional scaling (NMDS) analysis was used to visualize these results.

To test hypothesis 3, we identified taxa that either increased or decreased in relative abundance through time using multivariate generalized linear models (Wang *et al*., 2012). First, we filtered the OTU table to OTUs that were confidently assigned a genus‐level taxonomic identity (RDP classifier confidence threshold >0.7). OTUs within each genus were then aggregated by summing their abundances in each sample. We used negative binomial MV‐GLMs to account for overdispersion, and time was specified as a continuous variable (e.g. number of weeks). Likelihood ratio tests were used to test the statistical importance of time (number of weeks), brine origin, and sample type in predicting the relative abundance of each genus.

All graphics were created using the ‘ggplot2’ package (Wickham, 2009). Note that we report the results of all null hypothesis tests in terms of statistical clarity, rather than significance, in accordance with Dushoff and colleagues (2019).

## Supporting information

**Fig. S1.** Location map and local map of Trapani Salterns, Sicily.
**Fig. S2.** The taxonomic composition of the archaeal community from *in situ* brine and halite samples collected from pond 1. Low abundance genera comprising <0.1% of the total community are grouped as ‘Other’.
**Fig. S3.** The relative abundance of Archaea that showed statistically clear (*p* < 0.05) temporal changes in abundance across the duration of the succession experiment (21 weeks).
