# Supplementary material for: Extremely halophilic archaeal communities are resilient to short‐term entombment in halite
Source: Environ Microbiol. 2020 Jan 14;23(7):3370–83. doi: 10.1111/1462-2920.14913 (PMC8359394; doi:10.1111/1462-2920.14913)
Supplement: Supplementary file 1 — Fig. S1. Location map and local map of Trapani Salterns, Sicily. Fig. S2. The taxonomic composition of the archaeal community from in situ brine and halite samples collected from pond 1. Low abundance genera comprising <0.1% of the total community are grouped as ‘Other’. Fig. S3. The relative abundance of Archaea that showed statistically clear (p < 0.05) temporal changes in abundance across the duration of the succession experiment (21 weeks). [file EMI-23-3370-s001.docx]

**Supplementary Information to “Extremely Halophilic Archaeal Communities Are Resilient to Short-Term Entombment in Halite”**

Tom JC Huby^1†^, Dave R Clark^1†^, Boyd A McKew^1^, Terry J McGenity^1^

^1^School of Life Sciences, University of Essex, Colchester, Essex, UK

^†^ These authors contributed equally to this work.

**
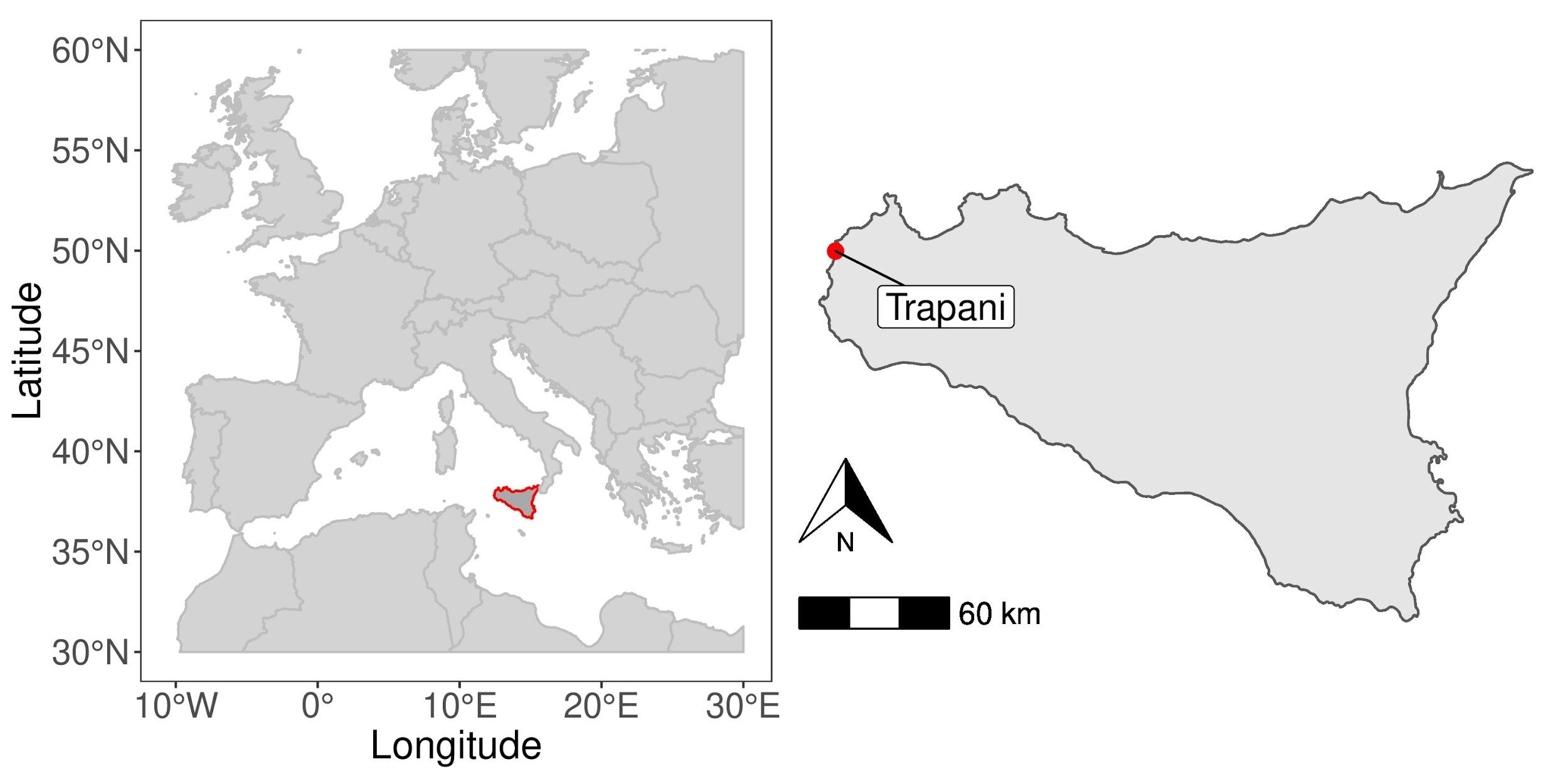
**

**Figure S1.** Location map and local map of Trapani Salterns, Sicily.


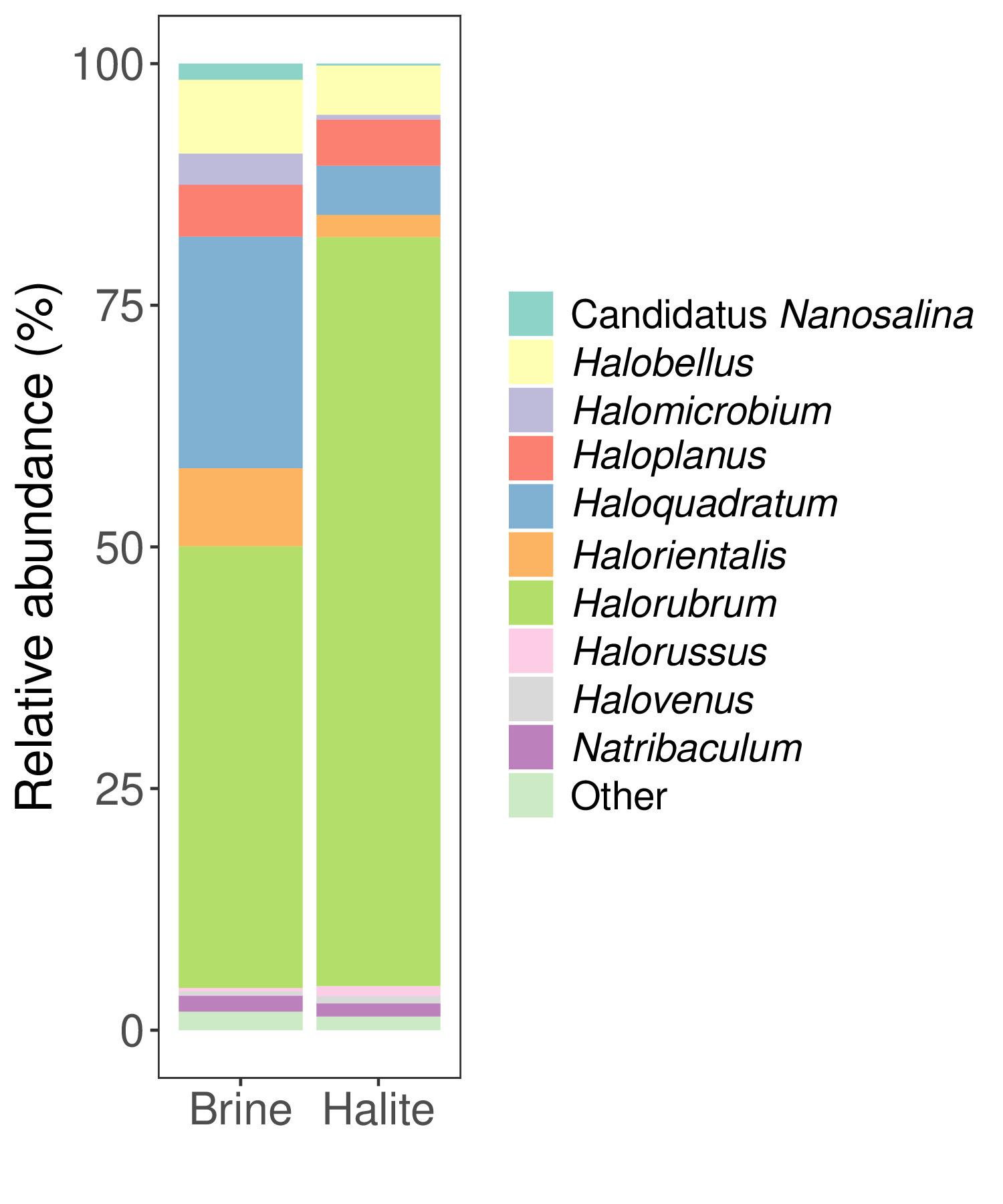


**Figure S2.** The taxonomic composition of the archaeal community from *in-situ* brine and halite samples collected from Pond 1. Low abundance genera comprising < 0.1% of the total community are grouped as “Other”.


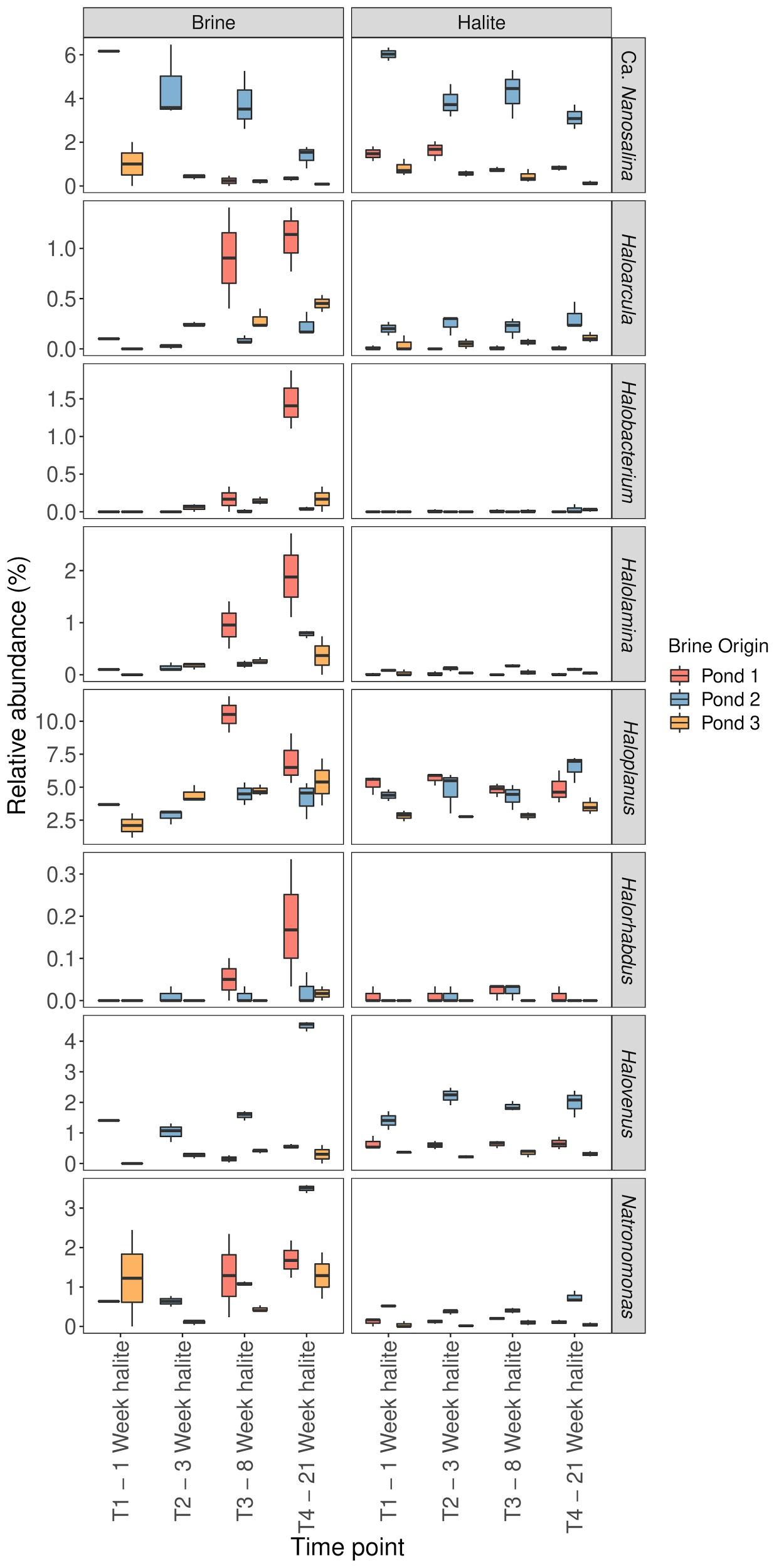


**Figure S3.** The relative abundance of Archaea that showed statistically clear (*P* < 0.05) temporal changes in abundance across the duration of the succession experiment (21 weeks).
